# Kernel Estimation of Cumulative Residual Tsallis Entropy and Its Dynamic Version under *ρ*-Mixing Dependent Data

**DOI:** 10.3390/e24010009

**Published:** 2021-12-21

**Authors:** Muhammed Rasheed Irshad, Radhakumari Maya, Francesco Buono, Maria Longobardi

**Affiliations:** 1 Department of Statistics, Cochin University of Science and Technology, Cochin 682 022, India; irshadmr@cusat.ac.in; 2 Department of Statistics, Govt. College for Women, Trivandrum 695 014, India; publicationsofmaya@gmail.com; 3 Dipartimento di Matematica e Applicazioni “Renato Caccioppoli”, Università degli Studi di Napoli Federico II, 80138 Naples, Italy; francesco.buono3@unina.it; 4 Dipartimento di Biologia, Università degli Studi di Napoli Federico II, 80138 Naples, Italy

**Keywords:** cumulative residual Tsallis entropy, dynamic cumulative residual Tsallis entropy, kernel estimator, ρ-mixing, simulation, 62B10, 62G20, 94A17

## Abstract

Tsallis introduced a non-logarithmic generalization of Shannon entropy, namely Tsallis entropy, which is non-extensive. Sati and Gupta proposed cumulative residual information based on this non-extensive entropy measure, namely cumulative residual Tsallis entropy (CRTE), and its dynamic version, namely dynamic cumulative residual Tsallis entropy (DCRTE). In the present paper, we propose non-parametric kernel type estimators for CRTE and DCRTE where the considered observations exhibit an ρ-mixing dependence condition. Asymptotic properties of the estimators were established under suitable regularity conditions. A numerical evaluation of the proposed estimator is exhibited and a Monte Carlo simulation study was carried out.

## 1. Introduction

Shannon [1] made his signature in statistics by introducing the concept of entropy, a measure of disorder in probability distribution. Associated with an absolutely continuous random variable *X* with probability density function (pdf) f(x), cumulative distribution function (cdf) F(x) and survival function (sf) $\bar{F}\left(x\right)=1-F\left(x\right)$, Shannon entropy is defined as

(1) $$\zeta \left(X\right)=-\int_{0}^{+\infty }f(x)\log f(x)dx,$$

where log(·) is the natural logarithm with standard convention 0log0=0. Nowadays, this measure has gained a peculiar place in sciences such as physics, chemistry, computer sciences, wavelet analysis, image recognition and fuzzy sets. Following the pioneering work of Shannon, the available literature has generated a significant amount of papers related to it, obtained by incorporating some additional parameters which make these entropies sensitive to different the shapes of probability distributions.

A vital generalization of Shannon entropy is Tsallis entropy, which was first introduced by Havrda and Charvát [2] in the status of cybernetics theory. Then, Tsallis [3] exploited its non-extensive features and described its paramount importance in physics. In parallel to Shannon entropy, it measures the disorder in macroscopic systems. For an absolutely continuous random variable *X* with pdf f(x), the Tsallis entropy of order α is defined as

(2) $$\tau_{\alpha }\left(X\right)=\frac{1}{\alpha -1}\left(1-E[{\left(f\left(X\right)\right)}^{\alpha -1}]\right)=\frac{1}{\alpha -1}\left(1-\int_{0}^{+\infty }{\left(f\left(x\right)\right)}^{\alpha }dx\right),\;\alpha \neq 1,\alpha >0.$$

when α→ 1, $\tau_{\alpha }\left(X\right)\to \zeta \left(X\right)$. Tsallis’s idea was to bestow a new formula instead of a classical logarithm used in Shannon entropy. Tsallis entropy is relevant in various fields of science; it is used in a broad range of contexts in physics science such as: statistical physics [4]; astrophysics [5]; turbulence [6]; inverse problems [7]; or quantum physics [8]. Tsallis entropy is applied in the description of the fluctuation of magnetic field in solar wind, in mammograms and in the analysis of magnetic resonance imaging (MRI) (as can be seen in Cartwright [9]). In recent years, this entropy has prompted many authors to define new discrimination measures as well as dual versions of entropy measures (see [10]).

Rao et al. [11] proposed another measure of uncertainty, called cumulative residual entropy (CRE), which is obtained by writing a survival function in place of pdf in (1) and is given by

(3) $$\nu \left(X\right)=-\int_{0}^{+\infty }\bar{F}\left(x\right)\log\bar{F}\left(x\right)dx.$$

The basic idea in this choice is that, in many situations, we prefer cumulative distribution function (cdf) over pdf. Moreover, a cdf exists in situations in which density does not exist such as in the case of a mixture density, combination of Gaussians and delta functions. The CRE is specifically applicable to describe the information in problems related to aging properties in reliability theory based on the mean residual life function. For other variants of CRE, one may refer to Rao [12], Psarrakos and Toomaj [13] and the references therein.

As in the scenario of introducing the concept of CRE, Sati and Gupta [14] introduced cumulative residual Tsallis entropy (CRTE) of order α, which is defined as

(4) $$\eta_{\alpha }\left(X\right)=\frac{1}{\alpha -1}\left(1-\int_{0}^{+\infty }{\left(\bar{F}\left(x\right)\right)}^{\alpha }dx\right),\;\;\alpha \neq 1,\alpha >0.$$

when α→ 1, $\eta_{\alpha }\left(X\right)\to \nu \left(X\right)$. Since $\eta_{\alpha }\left(X\right)$ is not applicable to a system that has survived for some units of time *t*, Sati and Gupta [14] proposed a dynamic version of CRTE based on the random variable $X_{t}=\left[X-t|X>t\right]$ whose definition is given below.

The dynamic cumulative residual Tsallis entropy (DCRTE) of order α is defined as

(5) $$\eta_{\alpha }\left(X;t\right)=\frac{1}{\alpha -1}\left(1-\int_{t}^{+\infty }{\left(\frac{\bar{F}\left(x\right)}{\bar{F}\left(t\right)}\right)}^{\alpha }dx\right),\;\;\alpha \neq 1,\alpha >0,$$

where $\bar{F}\left(\cdot \right)$ is the sf of *X*. Khammar and Jahanshahi [15] developed a weighted form of CRTE and DCRTE and discussed many of its reliability properties. Sunoj et al. [16] discussed a quantile-based study of CRTE and certain characterization results using order statistics. Mohamed [17] studied the CRTE and DCRTE of concomitants of generalized order statistics. Recently, Toomaj and Atabay [18] elaborately elucidated certain new results based on CRTE. The huge increase in the number of articles on CRTE and DCRTE shows the remarkable importance of both these measures from a theoretical and applied perspective especially in the physical context. As far as statistical inferential aspects are concerned, to the best of our knowledge, not even a single work has been performed to date in the available literature. Hence, in this work, our main objective is to propose non-parametric estimators for CRTE and DCRTE using kernel type estimation where the observations under considerations are exhibiting some mode of dependence. Practically, it seems more realistic to replace the independence with some mode of dependence.

The study of non-parametric density estimation in the case of dependent data was started decades back. Bradley [19] discussed the weak consistency and asymptotic normality of the kernel density estimator $f_{n}$ under ρ-mixing. Masry [20] established a non-parametric recursive density estimator in the α-mixing context and studied some of its properties. Masry and Györfi [21] established the strong consistency of recursive density estimator under ρ-mixing. Boente [22] discussed the strong consistency of the non-parametric density estimator under ϕ-mixing and α-mixing processes. The mixing coefficients α-mixing, ϕ-mixing and ρ-mixing are defined by Rosenblatt [23], Ibragimov [24] and Kolmogorov and Rozanov [25], respectively. For more properties of the different mixing coefficients, see Bradley [26].

Rajesh et al. [27] discussed the local linear estimation of the residual entropy function of conditional distributions where underlying observations are assumed to be ρ-mixing. The kernel estimation of the Mathai–Haubold entropy function under α-mixing dependence conditions were studied by Maya and Irshad [28]. Recently, non-parametric estimation using kernel type estimation under α-mixing dependence conditions of residual extropy, past extropy and negative cumulative residual extropy functions were studied by Maya and Irshad [29], Irshad and Maya [30] and Maya et al. [31], respectively. Compared to the α-mixing, ρ-mixing is stronger, as can be seen in Kolmogorov and Rozanov [25]. In this work, we propose non-parametric estimators of CRTE and DCRTE using kernel type estimation based on the assumption that underlying lifetimes are assumed to be ρ-mixing.

**Definition** **1.** *Let (Ω,F,P) be a probability space and $F_{i}^{k}$ be the σ-algebra of events obtained by the random variables $\left\{X_{j};i\leq j\leq k\right\}$. The stationary process $\left\{X_{j}\right\}$ is said to be asymptotically uncorrelated if*

(6) $$\underset{V\in L_{2}\left(F_{i+k}^{+\infty }\right)}{\underset{U\in L_{2}\left(F_{-\infty }^{i}\right)}{\sup}}\frac{\left|cov(U,V)\right|}{\sqrt{var(U)}\sqrt{var(V)}}=\rho \left(k\right)↓0$$

*as k→+∞, where $L_{2}\left(F_{a}^{b}\right)$ denotes the collection of all second-order random variables measurable with respect to $F_{a}^{b}$, ρ(k) is called the maximal correlation coefficient or ρ-mixing coefficient (as can be seen in Kolmogorov and Rozanov [25]).*

The rest of the paper is structured as follows. In Section 2, we propose non-parametric kernel type estimators for CRTE and DCRTE. Section 3 contains the expression for the bias and variances of the estimators proposed for CRTE and DCRTE and examines its consistency property. The mean consistently integrated in the quadratic mean and asymptotic normality of the proposed estimators are also discussed here in the form of several theorems. A numerical study on the asymptotic normality of the proposed estimators is given in Section 4.

## 2. Estimation

In this section, we propose non-parametric estimators for CRTE and DCRTE functions. Let $\left\{X_{i}\right\}$ be a strictly stationary process with univariate probability density function f(x). Note that $X{}_{i}$’s need not be mutually independent, that is, the lifetimes are assumed to be ρ-mixing. Wegman and Davies [32] introduced a recursive density estimator of f(x) given by

(7) $$f_{n}^{*}\left(x\right)=\frac{1}{n\sqrt{b_{n}}}\sum_{j=1}^{n}b_{j}^{-\frac{1}{2}}K\left(\frac{x-X_{j}}{b_{j}}\right),$$

where K(x) satisfies the following conditions:

$$\begin{array}{cc} \; & \sup|K\left(x\right)|<+\infty ,\;\;\;\int_{-\infty }^{+\infty }\left|K\left(x\right)\right|dx<+\infty ,\\ \; & \lim_{|x|\to +\infty }\left|xK\left(x\right)\right|=0,\;\;\;\int_{-\infty }^{+\infty }K\left(x\right)dx=1. \end{array}$$

The bandwidth parameter $b_{n}$ satisfies $b_{n}\to 0$ and $nb_{n}\to +\infty$ as n→+∞. Let *x* be a point of continuity of *f*. Suppose *f* is (r+1) times continuously differentiable at the point *x* such that:

$$\underset{u}{\sup}\left|f^{\left(r+1\right)}\left(u\right)\right|=M<+\infty .$$

Assume that:

$$\int_{-\infty }^{+\infty }{\left|u\right|}^{j}\left|K\left(u\right)\right|du<+\infty ,\;\;j=1,2,\cdots ,r+1,$$

and the bandwidth parameter $b_{n}$ satisfies:

$$\frac{1}{n}\sum_{j=1}^{n}{\left(\frac{b_{j}}{b_{n}}\right)}^{l+\frac{1}{2}}\to \beta_{l+\frac{1}{2}}<+\infty ,\mathrm{as}n\to +\infty ,\;\;l=0,1,2,\cdots ,r+1.$$

Then the mean and variance of $f_{n}^{*}\left(x\right)$ are given by (see, Masry [20])

(8) $$E\overset{}{}\left(f_{n}^{*}\left(x\right)\right)⋍\beta_{0.5}\left(f\left(x\right)+\frac{b_{n}^{2}c_{2}}{2\beta_{0.5}}f^{\left(2\right)}\left(x\right)\beta_{2.5}\right)$$

and:

(9) $$Var\overset{}{}\left(f_{n}^{*}\left(x\right)\right)⋍\frac{f(x)}{nb{}_{n}}C_{K},$$

where $c_{2}=\int_{-\infty }^{+\infty }u^{2}K\left(u\right)\;du$ and $C_{K}=\int_{-\infty }^{+\infty }K^{2}\left(u\right)\;du$. Equation (8) implies that $f_{n}^{*}\left(x\right)$ is not an asymptotically unbiased estimator of f(x). By simple scaling, we can find an asymptotically unbiased estimator of f(x) given by

$${\hat{f}}_{n}\left(x\right)=\frac{f_{n}^{*}\left(x\right)}{\beta_{0.5}}.$$

The bias and variance of ${\hat{f}}_{n}\left(x\right)$ are given by (see, Masry [20])

(10) $$Bias\overset{}{}\left({\hat{f}}_{n}\left(x\right)\right)⋍\frac{b_{n}^{2}c_{2}}{2\beta_{0.5}}f^{\left(2\right)}\left(x\right)\beta_{2.5}$$

and:

(11) $$Var\overset{}{}\left({\hat{f}}_{n}\left(x\right)\right)⋍\frac{f(x)}{nb{}_{n}{\beta_{0.5}}^{2}}C_{K}.$$

We propose kernel estimators for CRTE and DCRTE functions that are, respectively, given by

(12) $${\hat{\eta }}_{\alpha }\left(X\right)=\frac{1}{\alpha -1}\left\{1-\int_{0}^{+\infty }{\hat{\bar{F}}}_{n}^{\alpha }\left(x\right)dx\right\}$$

and:

(13) $${\hat{\eta }}_{\alpha }\left(X;t\right)=\frac{1}{\alpha -1}\left\{1-\frac{\int_{t}^{+\infty }{\hat{\bar{F}}}_{n}^{\alpha }\left(x\right)dx}{{\hat{\bar{F}}}_{n}^{\alpha }\left(t\right)}\right\},$$

where:

$$\begin{array}{cc} {\hat{\bar{F}}}_{n}\left(t\right) & =\int_{t}^{+\infty }{\hat{f}}_{n}\left(x\right)dx. \end{array}$$

is the non-parametric estimator of survival function $\bar{F}\left(t\right)$.

## 3. Asymptotic Results

Here, we propose the expression for bias, variance and certain asymptotic results of the proposed estimators.

**Theorem** **1.** *Let K(x) be a kernel satisfying the assumptions given in Section 2. Under ρ-mixing dependence conditions, we have:*

(14) $$Bias\left({\hat{\bar{F}}}_{n}\left(t\right)\right)⋍\frac{\beta_{2.5}}{2\beta_{0.5}}b_{n}^{2}c_{2}\int_{t}^{+\infty }f^{\left(2\right)}\left(x\right)\;dx,$$

*and:*

(15) $$Var\left({\hat{\bar{F}}}_{n}\left(t\right)\right)⋍\frac{1}{nb_{n}\beta_{0.5}^{2}}C_{K}\int_{t}^{+\infty }f\left(x\right)\;dx.$$

**Proof.**  The proof of the theorem is similar to the proof of bias and variance of ${\hat{f}}_{n}\left(x\right)$ given in Masry [20] and hence omitted. □

**Theorem** **2.** *Suppose ${\hat{\eta }}_{\alpha }\left(X\right)$ is a non-parametric estimator of CRTE defined in (12) and ${\hat{\eta }}_{\alpha }\left(X;t\right)$ is a non-parametric estimator of DCRTE defined in (13). Then, for $\alpha >\frac{1}{2}$ and α≠1:*

*1*. *${\hat{\eta }}_{\alpha }\left(X\right)$ is a consistent estimator of $\eta_{\alpha }\left(X\right)$;*
*2*. *${\hat{\eta }}_{\alpha }\left(X;t\right)$ is a consistent estimator of $\eta_{\alpha }\left(X;t\right)$.*

**Proof.**  *1.* By using the Taylor’s series expansion, we obtain:

$$\int_{0}^{+\infty }{\hat{\bar{F}}}_{n}^{\alpha }\left(x\right)dx⋍\int_{0}^{+\infty }{\bar{F}}^{\alpha }\left(x\right)dx+\alpha \int_{0}^{+\infty }{\bar{F}}^{\alpha -1}\left(x\right)\left({\hat{\bar{F}}}_{n}\left(x\right)-\bar{F}\left(x\right)\right)dx.$$

Using the above equation, the bias and the variance of $\int_{0}^{+\infty }{\hat{\bar{F}}}_{n}^{\alpha }\left(x\right)dx$ are given by

(16) $$Bias\left(\int_{0}^{+\infty }{\hat{\bar{F}}}_{n}^{\alpha }\left(x\right)dx\right)⋍\frac{\alpha \beta_{2.5}}{2\beta_{0.5}}b_{n}^{2}c_{2}\int_{0}^{+\infty }\left(\int_{x}^{+\infty }f^{\left(2\right)}\left(y\right)\;dy\right){\bar{F}}^{\alpha -1}\left(x\right)dx$$

and:

(17) $$Var\left(\int_{0}^{+\infty }{\hat{\bar{F}}}_{n}^{\alpha }\left(x\right)dx\right)⋍\frac{\alpha^{2}}{nb_{n}\beta_{0.5}^{2}}C_{K}\int_{0}^{\infty }{\bar{F}}^{2\alpha -1}\left(x\right)dx.$$

The corresponding MSE is given by

(18) $$\begin{array}{cc} MSE\left(\int_{0}^{+\infty }{\hat{\bar{F}}}_{n}^{\alpha }\left(x\right)dx\right) & ⋍{\left(\frac{\alpha \beta_{2.5}}{2\beta_{0.5}}b_{n}^{2}c_{2}\int_{0}^{+\infty }\left(\int_{x}^{+\infty }f^{\left(2\right)}\left(y\right)\;dy\right){\bar{F}}^{\alpha -1}\left(x\right)dx\right)}^{2}\\ \; & +\frac{\alpha^{2}}{nb_{n}\beta_{0.5}^{2}}C_{K}\int_{0}^{+\infty }{\bar{F}}^{2\alpha -1}\left(x\right)dx. \end{array}$$

From (18), as n→+∞:

$$MSE\left(\int_{0}^{+\infty }{\hat{\bar{F}}}_{n}^{\alpha }\left(x\right)dx\right)\to 0.$$

Therefore:

$${\hat{\eta }}_{\alpha }\left(X\right)=\frac{1}{\alpha -1}\left\{1-\int_{0}^{+\infty }{\hat{\bar{F}}}_{n}^{\alpha }\left(x\right)dx\right\}\overset{p}{\to }\frac{1}{\alpha -1}\left\{1-\int_{0}^{+\infty }{\bar{F}}^{\alpha }\left(x\right)dx\right\}=\eta_{\alpha }\left(X\right).$$

Hence, ${\hat{\eta }}_{\alpha }\left(X\right)$ is a consistent estimator (in the probability sense) of $\eta_{\alpha }\left(X\right)$.

*2.* By using Taylor’s series expansion, the expressions for the bias of $\int_{t}^{+\infty }{\hat{\bar{F}}}_{n}^{\alpha }\left(x\right)dx$ and ${\hat{\bar{F}}}_{n}^{\alpha }\left(t\right)$ are given by

(19) $$\begin{array}{ccc} Bias\left(\int_{t}^{+\infty }{\hat{\bar{F}}}_{n}^{\alpha }\left(x\right)dx\right) & ⋍ & \frac{\alpha \beta_{2.5}}{2\beta_{0.5}}b_{n}^{2}c_{2}\int_{t}^{+\infty }\left(\int_{x}^{+\infty }f^{\left(2\right)}\left(y\right)\;dy\right){\bar{F}}^{\alpha -1}\left(x\right)dx, \end{array}$$

(20) $$\begin{array}{ccc} Bias\left({\hat{\bar{F}}}_{n}^{\alpha }\left(t\right)\right) & ⋍ & \frac{\alpha \beta_{2.5}}{2\beta_{0.5}}b_{n}^{2}c_{2}{\bar{F}}^{\alpha -1}\left(t\right)\int_{t}^{+\infty }f^{\left(2\right)}\left(y\right)\;dy, \end{array}$$

whereas the variances are given by

(21) $$\begin{array}{ccc} Var\left(\int_{t}^{+\infty }{\hat{\bar{F}}}_{n}^{\alpha }\left(x\right)dx\right) & ⋍ & \frac{\alpha^{2}}{nb_{n}\beta_{0.5}^{2}}C_{K}\int_{t}^{+\infty }{\bar{F}}^{2\alpha -1}\left(x\right)dx, \end{array}$$

(22) $$\begin{array}{ccc} Var\left({\hat{\bar{F}}}_{n}^{\alpha }\left(t\right)\right) & ⋍ & \frac{\alpha^{2}}{nb_{n}\beta_{0.5}^{2}}C_{K}{\bar{F}}^{2\alpha -1}\left(t\right). \end{array}$$

The corresponding MSE’s are given by

(23) $$\begin{array}{cc} MSE\left(\int_{t}^{+\infty }{\hat{\bar{F}}}_{n}^{\alpha }\left(x\right)dx\right) & ⋍{\left(\frac{\alpha \beta_{2.5}}{2\beta_{0.5}}b_{n}^{2}c_{2}\int_{t}^{+\infty }\left(\int_{x}^{+\infty }f^{\left(2\right)}\left(y\right)\;dy\right){\bar{F}}^{\alpha -1}\left(x\right)dx\right)}^{2}\\ \; & +\frac{\alpha^{2}}{nb_{n}\beta_{0.5}^{2}}C_{K}\int_{t}^{+\infty }{\bar{F}}^{2\alpha -1}\left(x\right)dx \end{array}$$

and:

(24) $$\begin{array}{cc} MSE\left({\hat{\bar{F}}}_{n}^{\alpha }\left(t\right)\right) & ⋍{\left(\frac{\alpha \beta_{2.5}}{2\beta_{0.5}}b_{n}^{2}c_{2}{\bar{F}}^{\alpha -1}\left(t\right)\int_{t}^{+\infty }f^{\left(2\right)}\left(y\right)\;dy\right)}^{2}\\ \; & +\frac{\alpha^{2}}{nb_{n}\beta_{0.5}^{2}}C_{K}{\bar{F}}^{2\alpha -1}\left(t\right). \end{array}$$

From (23) and (24), as n→+∞:

$$MSE\left(\int_{t}^{+\infty }{\hat{\bar{F}}}_{n}^{\alpha }\left(x\right)dx\right)\to 0,$$

and:

$$MSE\left({\hat{\bar{F}}}_{n}^{\alpha }\left(t\right)\right)\to 0.$$

Therefore:

$${\hat{\eta }}_{\alpha }\left(X;t\right)=\frac{1}{\alpha -1}\left\{1-\frac{\int_{t}^{+\infty }{\hat{\bar{F}}}_{n}^{\alpha }\left(x\right)dx}{{\hat{\bar{F}}}_{n}^{\alpha }\left(t\right)}\right\}\overset{p}{\to }\frac{1}{\alpha -1}\left\{1-\frac{\int_{t}^{+\infty }{\bar{F}}^{\alpha }\left(x\right)dx}{{\bar{F}}^{\alpha }\left(t\right)}\right\}=\eta_{\alpha }\left(X;t\right).$$

Hence, ${\hat{\eta }}_{\alpha }\left(X;t\right)$ is a consistent estimator (in the probability sense) of $\eta_{\alpha }\left(X;t\right)$. □

**Proposition** **1.** *Let K(x) be a kernel satisfying the conditions given in Section 2. Then, the estimation error for DCRTE defined in (13) is given by*

(25) $${\hat{\eta }}_{\alpha }\left(X;t\right)-\eta_{\alpha }\left(X;t\right)⋍\frac{-1}{\left(\alpha -1\right)A_{\alpha }\left(t\right)}\left({\hat{M}}_{\alpha }\left(t\right)-{\hat{A}}_{\alpha }\left(t\right)\frac{M_{\alpha }\left(t\right)}{A_{\alpha }\left(t\right)}\right),$$

*where ${\hat{M}}_{\alpha }\left(t\right)=\int_{t}^{+\infty }{\hat{\bar{F}}}_{n}^{\alpha }\left(x\right)dx$, $M_{\alpha }\left(t\right)=\int_{t}^{+\infty }{\bar{F}}^{\alpha }\left(x\right)dx$, ${\hat{A}}_{\alpha }\left(t\right)={\hat{\bar{F}}}_{n}^{\alpha }\left(t\right)$ and $A_{\alpha }\left(t\right)={\bar{F}}^{\alpha }\left(t\right).$*

**Proof.**  We have:

(26) $$\begin{array}{cc} \frac{{\hat{M}}_{\alpha }\left(t\right)}{{\hat{A}}_{\alpha }\left(t\right)}-\frac{M_{\alpha }\left(t\right)}{A_{\alpha }\left(t\right)} & =\frac{1}{A_{\alpha }\left(t\right)}\left({\hat{M}}_{\alpha }\left(t\right)-{\hat{A}}_{\alpha }\left(t\right)\frac{M_{\alpha }\left(t\right)}{A_{\alpha }\left(t\right)}\right)\left(\frac{A_{\alpha }\left(t\right)}{{\hat{A}}_{\alpha }\left(t\right)}-1\right)+\\ \; & \frac{1}{A_{\alpha }\left(t\right)}\left({\hat{M}}_{\alpha }\left(t\right)-{\hat{A}}_{\alpha }\left(t\right)\frac{M_{\alpha }\left(t\right)}{A_{\alpha }\left(t\right)}\right)\\ \; & =\frac{1}{A_{\alpha }\left(t\right)}\left({\hat{M}}_{\alpha }\left(t\right)-{\hat{A}}_{\alpha }\left(t\right)\frac{M_{\alpha }\left(t\right)}{A_{\alpha }\left(t\right)}\right)\left(1+O_{p}\left(1\right)\right) \end{array}$$

with $\left(\frac{A_{\alpha }\left(t\right)}{{\hat{A}}_{\alpha }\left(t\right)}-1\right)=O_{p}\left(1\right)$, since ${\hat{A}}_{\alpha }\left(t\right)\overset{p}{\to }A_{\alpha }\left(t\right)$.

Therefore:

(27) $$\begin{array}{cc} \frac{{\hat{M}}_{\alpha }\left(t\right)}{{\hat{A}}_{\alpha }\left(t\right)}-\frac{M_{\alpha }\left(t\right)}{A_{\alpha }\left(t\right)} & ⋍\frac{1}{A_{\alpha }\left(t\right)}\left({\hat{M}}_{\alpha }\left(t\right)-{\hat{A}}_{\alpha }\left(t\right)\frac{M_{\alpha }\left(t\right)}{A_{\alpha }\left(t\right)}\right). \end{array}$$

We have:

(28) $${\hat{\eta }}_{\alpha }\left(X;t\right)-\eta_{\alpha }\left(X;t\right)⋍\frac{-1}{\left(\alpha -1\right)}\left(\frac{{\hat{M}}_{\alpha }\left(t\right)}{{\hat{A}}_{\alpha }\left(t\right)}-\frac{M_{\alpha }\left(t\right)}{A_{\alpha }\left(t\right)}\right).$$

By substituting (27) in (28), we obtain (25). □

**Theorem** **3.** *Suppose ${\hat{\eta }}_{\alpha }\left(X\right)$ is a non-parametric estimator of CRTE defined in (12) and ${\hat{\eta }}_{\alpha }\left(X;t\right)$ is a non-parametric estimator of DCRTE defined in (13). Then, the biases of ${\hat{\eta }}_{\alpha }\left(X\right)$ and ${\hat{\eta }}_{\alpha }\left(X;t\right)$ are given as*

(29) $$\begin{array}{ccc} Bias({\hat{\eta }}_{\alpha }\left(X\right)) & ⋍ & \frac{-\alpha }{\left(\alpha -1\right)}\frac{b_{n}^{2}c_{2}\beta_{2.5}}{2\beta_{0.5}}\left\{\int_{0}^{+\infty }{\bar{F}}^{\alpha -1}\left(x\right)\left(\int_{x}^{+\infty }f^{\left(2\right)}\left(y\right)\;dy\right)dx\right\}, \end{array}$$

(30) $$\begin{array}{ccc} Bias({\hat{\eta }}_{\alpha }\left(X;t\right)) & ⋍ & \frac{\alpha }{\left(\alpha -1\right)}\frac{\beta_{2.5}}{2\beta_{0.5}}\frac{b_{n}^{2}c_{2}}{{\bar{F}}^{\alpha }\left(t\right)}\left\{\frac{\int_{t}^{+\infty }{\bar{F}}^{\alpha }\left(x\right)dx}{\bar{F}\left(t\right)}\int_{t}^{+\infty }f^{\left(2\right)}\left(y\right)\;dy\right.\\ & & \left.\;\;-\int_{t}^{+\infty }{\bar{F}}^{\alpha -1}\left(x\right)\left(\int_{x}^{+\infty }f^{\left(2\right)}\left(y\right)\;dy\right)dx\right\}, \end{array}$$

*and the variances are given for $\alpha >\frac{1}{2}$ as*

(31) $$\begin{array}{ccc} Var({\hat{\eta }}_{\alpha }\left(X\right)) & ⋍ & \frac{\alpha^{2}}{{\left(\alpha -1\right)}^{2}nb_{n}\beta_{0.5}^{2}}C_{K}\left\{\int_{0}^{+\infty }{\bar{F}}^{2\alpha -1}\left(x\right)dx\right\}, \end{array}$$

(32) $$\begin{array}{ccc} Var({\hat{\eta }}_{\alpha }\left(X;t\right)) & ⋍ & \frac{\alpha^{2}}{{\left(\alpha -1\right)}^{2}nb_{n}\beta_{0.5}^{2}{\bar{F}}^{2\alpha }\left(t\right)}C_{K}\left\{\int_{t}^{+\infty }{\bar{F}}^{2\alpha -1}\left(x\right)dx+\frac{{\left(\int_{t}^{+\infty }{\bar{F}}^{\alpha }\left(x\right)dx\right)}^{2}}{\bar{F}\left(t\right)}\right\}. \end{array}$$

**Proof.**  By using Equations (16) and (17), we obtain the bias and variance of ${\hat{\eta }}_{\alpha }\left(X\right)$ and by using Proposition 1 and Equations (19)–(22), we obtain the bias and variance of ${\hat{\eta }}_{\alpha }\left(X;t\right)$. □

**Theorem** **4.** *Suppose ${\hat{\eta }}_{\alpha }\left(X\right)$ is a non-parametric estimator of CRTE as defined in (12) and ${\hat{\eta }}_{\alpha }\left(X;t\right)$ is a non-parametric estimator of DCRTE as defined in (13). Then, for $\alpha >\frac{1}{2}$ and α≠1:*

*1*. *${\hat{\eta }}_{\alpha }\left(X\right)$ is integratedly uniformly consistent in the quadratic mean estimator of $\eta_{\alpha }\left(X\right)$;*
*2*. *${\hat{\eta }}_{\alpha }\left(X;t\right)$ is integratedly uniformly consistent in the quadratic mean estimator of $\eta_{\alpha }\left(X;t\right)$.*

**Proof.**  *1.* Consider the mean integrated squared error (MISE) of the estimator ${\hat{\eta }}_{\alpha }\left(X\right)$. That is:

(33) $$\begin{array}{cc} MISE({\hat{\eta }}_{\alpha }\left(X\right)) & =E\int_{0}^{+\infty }{\left[{\hat{\eta }}_{\alpha }\left(X\right)-\eta_{\alpha }\left(X\right)\right]}^{2}dx\\ \; & =\int_{0}^{+\infty }E{\left[{\hat{\eta }}_{\alpha }\left(X\right)-\eta_{\alpha }\left(X\right)\right]}^{2}dx\\ \; & =\int_{0}^{+\infty }\left[Var\left({\hat{\eta }}_{\alpha }\left(X\right)\right)+{\left[Bias\left({\hat{\eta }}_{\alpha }\left(X\right)\right)\right]}^{2}\right]dx\\ \; & =\int_{0}^{+\infty }MSE\left({\hat{\eta }}_{\alpha }\left(X\right)\right)dx. \end{array}$$

Using (29) and (31), we obtain:

(34) $$\begin{array}{ccc} MSE\left({\hat{\eta }}_{\alpha }\left(X\right)\right) & ⋍ & {\left(\frac{-\alpha }{\left(\alpha -1\right)}\frac{b_{n}^{2}c_{2}\beta_{2.5}}{2\beta_{0.5}}\left\{\int_{0}^{+\infty }{\bar{F}}^{\alpha -1}\left(x\right)\left(\int_{x}^{+\infty }f^{\left(2\right)}\left(y\right)\;dy\right)dx\right\}\right)}^{2}\\ & & +\frac{\alpha^{2}}{{\left(\alpha -1\right)}^{2}nb_{n}\beta_{0.5}^{2}}C_{K}\left\{\int_{0}^{+\infty }{\bar{F}}^{2\alpha -1}\left(x\right)dx\right\}. \end{array}$$

From (34), as n→+∞:

$$MSE\left({\hat{\eta }}_{\alpha }\left(X\right)\right)\to 0.$$

Therefore, from (33), we have:

(35) $$MISE\left({\hat{\eta }}_{\alpha }\left(X\right)\right)\to 0,\;as\;n\to +\infty .$$

From (35), we can say that ${\hat{\eta }}_{\alpha }\left(X\right)$ is integratedly uniformly consistent in quadratic mean estimator of $\eta_{\alpha }\left(X\right)$ (as can be seen in Wegman [33]).

*2.* Consider the MISE of the estimator ${\hat{\eta }}_{\alpha }\left(X;t\right)$—that is:

(36) $$\begin{array}{cc} MISE({\hat{\eta }}_{\alpha }\left(X;t\right)) & =E\int_{0}^{+\infty }{\left[{\hat{\eta }}_{\alpha }\left(X;t\right)-\eta_{\alpha }\left(X;t\right)\right]}^{2}dx\\ \; & =\int_{0}^{+\infty }E{\left[{\hat{\eta }}_{\alpha }\left(X;t\right)-\eta_{\alpha }\left(X;t\right)\right]}^{2}dx\\ \; & =\int_{0}^{+\infty }\left[Var\left({\hat{\eta }}_{\alpha }\left(X;t\right)\right)+{\left[Bias\left({\hat{\eta }}_{\alpha }\left(X;t\right)\right)\right]}^{2}\right]dx\\ \; & =\int_{0}^{+\infty }MSE\left({\hat{\eta }}_{\alpha }\left(X;t\right)\right)dx. \end{array}$$

Using (30) and (32), we obtain:

(37) $$\begin{array}{ccc} MSE\left({\hat{\eta }}_{\alpha }\left(X;t\right)\right) & ⋍ & \left(\frac{\alpha }{\left(\alpha -1\right)}\frac{\beta_{2.5}}{2\beta_{0.5}}\frac{b_{n}^{2}c_{2}}{{\bar{F}}^{\alpha }\left(t\right)}\left\{\frac{\int_{t}^{+\infty }{\bar{F}}^{\alpha }\left(x\right)dx}{\bar{F}\left(t\right)}\int_{t}^{+\infty }f^{\left(2\right)}\left(y\right)\;dy\right.\right.\\ & & {\left.\left.\;\;-\int_{t}^{+\infty }{\bar{F}}^{\alpha -1}\left(x\right)\left(\int_{x}^{+\infty }f^{\left(2\right)}\left(y\right)\;dy\right)dx\right\}\right)}^{2}+\\ & & \frac{\alpha^{2}}{{\left(\alpha -1\right)}^{2}nb_{n}\beta_{0.5}^{2}{\bar{F}}^{2\alpha }\left(t\right)}C_{K}\left\{\int_{t}^{+\infty }{\bar{F}}^{2\alpha -1}\left(x\right)dx+\frac{{\left(\int_{t}^{+\infty }{\bar{F}}^{\alpha }\left(x\right)dx\right)}^{2}}{\bar{F}\left(t\right)}\right\}. \end{array}$$

From (37), as n→+∞:

$$MSE\left({\hat{\eta }}_{\alpha }\left(X;t\right)\right)\to 0.$$

Therefore, from (36), we have:

(38) $$MISE\left({\hat{\eta }}_{\alpha }\left(X;t\right)\right)\to 0,\;as\;n\to +\infty .$$

From (38), we can say that ${\hat{\eta }}_{\alpha }\left(X;t\right)$ is integratedly uniformly consistent in the quadratic mean estimator of $\eta_{\alpha }\left(X;t\right)$ (as can be seen in Wegman [33]). □

**Theorem** **5.** *Suppose that ${\hat{\eta }}_{\alpha }\left(X\right)$ is a non-parametric estimator of CRTE defined in (12) with $\alpha >\frac{1}{2}$. Then, as n→+∞:*

(39) $${\left(nb_{n}\right)}^{\frac{1}{2}}\left\{\frac{{\hat{\eta }}_{\alpha }\left(X\right)-\eta_{\alpha }\left(X\right)}{\sigma_{\eta }}\right\}$$

*has a standard normal distribution where:*

(40) $$\sigma_{\eta }^{2}⋍\frac{\alpha^{2}}{{\left(\alpha -1\right)}^{2}\beta_{0.5}^{2}}C_{K}\left\{\int_{0}^{+\infty }{\bar{F}}^{2\alpha -1}\left(x\right)dx\right\}.$$

**Proof.**  (41) $$\begin{array}{cc} {\left(nb_{n}\right)}^{\frac{1}{2}}\left({\hat{\eta }}_{n}\left(X\right)-\eta \left(X\right)\right) & =\frac{-{\left(nb_{n}\right)}^{\frac{1}{2}}}{\left(\alpha -1\right)}\left\{\int_{0}^{+\infty }{\hat{\bar{F}}}_{n}^{\alpha }\left(x\right)dx-\int_{0}^{+\infty }{\bar{F}}^{\alpha }\left(x\right)dx\right\}\\ \; & ⋍\frac{-\alpha {\left(nb_{n}\right)}^{\frac{1}{2}}}{\left(\alpha -1\right)}\left\{\int_{0}^{+\infty }{\bar{F}}^{\alpha -1}\left(x\right)\left(\int_{x}^{+\infty }\left({\hat{f}}_{n}\left(y\right)-f\left(y\right)\right)dy\right)dx\right\}. \end{array}$$

By using the asymptotic normality of ${\hat{f}}_{n}\left(x\right)$ given in Masry [20], it is immediate that:

$${\left(nb_{n}\right)}^{\frac{1}{2}}\left\{\frac{{\hat{\eta }}_{\alpha }\left(X\right)-\eta_{\alpha }\left(X\right)}{\sigma_{\eta }}\right\}$$

is asymptotically normal with a mean of zero, variance of 1 and $\sigma_{\eta }^{2}$ given in (40). □

**Theorem** **6.** *Suppose that ${\hat{\eta }}_{\alpha }\left(X;t\right)$ is a non-parametric estimator of DCRTE $\eta_{\alpha }\left(X;t\right)$ defined in (13) with $\alpha >\frac{1}{2}$ and α≠1. Then, as n→+∞:*

(42) $${\left(nb_{n}\right)}^{\frac{1}{2}}\left\{\frac{{\hat{\eta }}_{\alpha }\left(X;t\right)-\eta_{\alpha }\left(X;t\right)}{\sigma_{\eta_{x}}}\right\}$$

*has a standard normal distribution where:*

(43) $$\sigma_{\eta_{x}}^{2}⋍\frac{\alpha^{2}}{{\left(\alpha -1\right)}^{2}\beta_{0.5}^{2}{\bar{F}}^{2\alpha }\left(t\right)}C_{K}\left\{\int_{t}^{+\infty }{\bar{F}}^{2\alpha -1}\left(x\right)dx+\frac{{\left(\int_{t}^{+\infty }{\bar{F}}^{\alpha }\left(x\right)dx\right)}^{2}}{\bar{F}\left(t\right)}\right\}.$$

**Proof.** 
(44) $$\begin{array}{cc} {\left(nb_{n}\right)}^{\frac{1}{2}}\left({\hat{\eta }}_{n}\left(X;t\right)-\eta \left(X;t\right)\right) & =\frac{-{\left(nb_{n}\right)}^{\frac{1}{2}}}{\left(\alpha -1\right)}\left\{\frac{\int_{t}^{+\infty }{\hat{\bar{F}}}_{n}^{\alpha }\left(x\right)dx}{{\hat{\bar{F}}}_{n}^{\alpha }\left(t\right)}-\frac{\int_{t}^{+\infty }{\bar{F}}^{\alpha }\left(x\right)dx}{{\bar{F}}^{\alpha }\left(t\right)}\right\}\\ \; & ⋍\frac{-\alpha {\left(nb_{n}\right)}^{\frac{1}{2}}}{\left(\alpha -1\right){\bar{F}}^{\alpha }\left(t\right)}\int_{t}^{+\infty }{\bar{F}}^{\alpha -1}\left(x\right)\left({\hat{\bar{F}}}_{n}^{\alpha }\left(x\right)-{\bar{F}}^{\alpha }\left(x\right)\right)dx\\ \; & =\frac{-\alpha {\left(nb_{n}\right)}^{\frac{1}{2}}}{\left(\alpha -1\right){\bar{F}}^{\alpha }\left(t\right)}\left\{\int_{t}^{+\infty }{\bar{F}}^{\alpha -1}\left(x\right)\left(\int_{x}^{+\infty }\left({\hat{f}}_{n}\left(y\right)-f\left(y\right)\right)dy\right)dx\right\}. \end{array}$$

By using the asymptotic normality of ${\hat{f}}_{n}\left(x\right)$ given in Masry [20], it is immediate that:

$${\left(nb_{n}\right)}^{\frac{1}{2}}\left\{\frac{{\hat{\eta }}_{\alpha }\left(X;t\right)-\eta_{\alpha }\left(X;t\right)}{\sigma_{\eta_{x}}}\right\}$$

is asymptotically normal with a mean of zero, variance of 1 and $\sigma_{\eta_{x}}^{2}$ given in (43). □

## 4. Numerical Evaluation of ${\hat{\eta }}_{\alpha }\left(X\right)$ and Monte Carlo Simulation

In this section, a numerical evaluation of ${\hat{\eta }}_{\alpha }\left(X\right)$ is given and a Monte Carlo simulation is carried out to support the asymptotic normality of the estimator given in (39). Let *X* be exponentially distributed with parameter λ (mean 1/λ). Then, the CRTE of order α of *X* is given by

(45) $$\eta_{\alpha }\left(X\right)=\frac{\lambda \alpha -1}{\lambda \alpha (\alpha -1)}.$$

In order to obtain the desired estimator, it is necessary to fix a function *K* and a sequence ${\left\{b_{n}\right\}}_{n\in N}$ which satisfy the assumptions given in Section 2. Here, we consider:

(46) $$\begin{array}{ccc} K(x) & = & \frac{1}{\sqrt{2\pi }}\exp\left(-\frac{x^{2}}{2}\right),\;\;x\in R \end{array}$$

(47) $$\begin{array}{ccc} b_{n} & = & \frac{1}{\sqrt{n}},\;\;n\in N. \end{array}$$

By using these assumptions, it readily follows that:

(48) $$\begin{array}{ccc} \beta_{0.5} & = & \frac{4}{3}, \end{array}$$

(49) $$\begin{array}{ccc} C_{K} & = & \frac{1}{2\sqrt{\pi }} \end{array}$$

(50) $$\begin{array}{ccc} \sigma_{\eta }^{2} & = & \frac{\alpha^{2}C_{K}}{{\left(\alpha -1\right)}^{2}\beta_{0.5}^{2}\lambda \left(2\alpha -1\right)}=\frac{9\alpha^{2}}{32\sqrt{\pi }{\left(\alpha -1\right)}^{2}\lambda \left(2\alpha -1\right)}, \end{array}$$

where $\alpha >\frac{1}{2}$ and α≠1.

**Figure 1:**
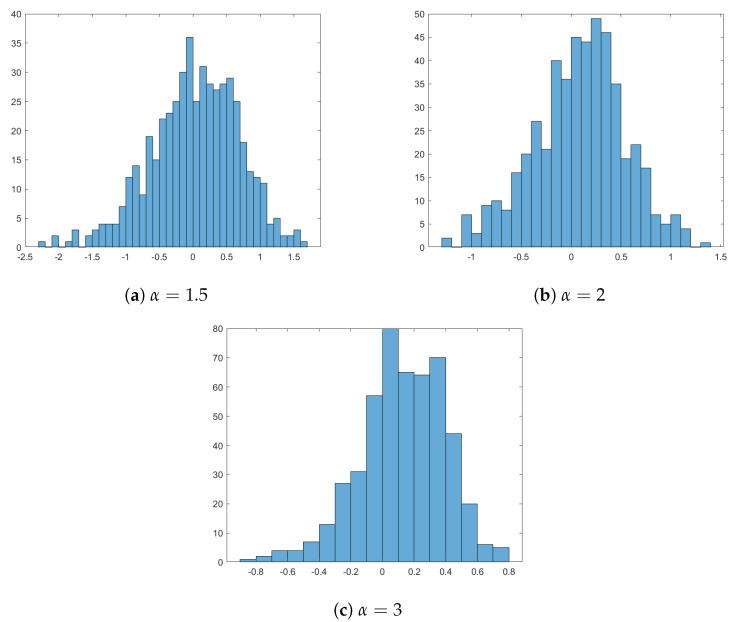
Histogram of (39) with parameters given in Section 4 and different choices of α.

To fix the ideas, consider n=50 and λ=1. Our goal is to check that:

$${\left(nb_{n}\right)}^{\frac{1}{2}}\left\{\frac{{\hat{\eta }}_{\alpha }\left(X\right)-\eta_{\alpha }\left(X\right)}{\sigma_{\eta }}\right\}$$

has a standard normal distribution. By using the function exprnd of *MATLAB*, 500 samples of size *n*, whose parent distribution is exponential with parameter 1, are generated. These data satisfy the assumption in (6). Hence, by using the fixed parameters, the functions ${\hat{f}}_{n}$ and ${\hat{\bar{F}}}_{n}$ are obtained for each sample and finally the kernel estimator of CRTE is computed. This procedure is repeated by choosing α=1.5, 2 and 3. Then, in order to check the asymptotic normality of the estimator in (39), the histogram in Figure 1 is displayed.

## 5. Conclusions

In this paper, non-parametric kernel type estimators for CRTE and DCRTE were proposed for observations which exhibit ρ-mixing dependence. The bias and the variance of the proposed estimators were evaluated. Moreover, it was proven that those estimators are consistent and a Monte Carlo simulation was carried out to show their asymptotic normality.

## Data Availability

Not applicable.

## Abbreviations

The following abbreviations are used in this manuscript:

cdf	cumulative distribution function
CRE	cumulative residual entropy
CRTE	cumulative residual Tsallis entropy
DCRTE	dynamic cumulative residual Tsallis entropy
MISE	mean integrated squared error
MSE	mean squared error
pdf	probability density function
sf	survival function
